# Metabolomics as a Prospective Tool for Soybean (*Glycine max*) Crop Improvement

**DOI:** 10.3390/cimb44090287

**Published:** 2022-09-12

**Authors:** Efficient Ncube, Keletso Mohale, Noluyolo Nogemane

**Affiliations:** Department of Agriculture and Animal Health, College of Agriculture and Environmental Sciences, University of South Africa, Science Campus, Private Bag x 6, Florida, Johannesburg 1710, South Africa

**Keywords:** biomarker, crop breeding, *Glycine max*, metabolomics, secondary metabolites

## Abstract

Global demand for soybean and its products has stimulated research into the production of novel genotypes with higher yields, greater drought and disease tolerance, and shorter growth times. Genetic research may be the most effective way to continue developing high-performing cultivars with desirable agronomic features and improved nutritional content and seed performance. Metabolomics, which predicts the metabolic marker for plant performance under stressful conditions, is rapidly gaining interest in plant breeding and has emerged as a powerful tool for driving crop improvement. The development of increasingly sensitive, automated, and high-throughput analytical technologies, paired with improved bioinformatics and other omics techniques, has paved the way for wide characterization of genetic characteristics for crop improvement. The combination of chromatography (liquid and gas-based) with mass spectrometry has also proven to be an indisputable efficient platform for metabolomic studies, notably plant metabolic fingerprinting investigations. Nevertheless, there has been significant progress in the use of nuclear magnetic resonance (NMR), capillary electrophoresis, and Fourier-transform infrared spectroscopy (FTIR), each with its own set of benefits and drawbacks. Furthermore, utilizing multivariate analysis, principal components analysis (PCA), discriminant analysis, and projection to latent structures (PLS), it is possible to identify and differentiate various groups. The researched soybean varieties may be correctly classified by using the PCA and PLS multivariate analyses. As metabolomics is an effective method for evaluating and selecting wild specimens with desirable features for the breeding of improved new cultivars, plant breeders can benefit from the identification of metabolite biomarkers and key metabolic pathways to develop new genotypes with value-added features.

## 1. Introduction

For centuries, mankind is entirely reliant on plants as the main source of nutrients. However, since the world population is increasing at a rapid rate, there is extreme pressure on the harvesting of health and nutritional contributing plants. Therefore, developing and implementing ways to reduce the impact of biotic and abiotic stresses on soybean yield and quality is critical for global food security [1,2].

Soja is a phylogenetic group that comprises wild soybean (*Glycine soja*), semi-wild soybean (*Glycine gracilis*), and cultivated soybean (*Glycine max*). Research has revealed that soybeans grown in the wild can better adapt to a variety of harsh conditions. Semi-wild soybean is a transition type in the Soja evolution, with a physiological metabolism similar to wild soybean and a phenotypic similar to cultivated soybean. Artificial selection and domestication have bred the cultivated soybean from wild soybean with origins from Asia [3].

Soybean, first grown in East Asia millennia years ago, is a vital source of nourishment for people all over the world, and it is widely regarded as a nutritious meal in many Asian countries. Humans have been growing and consuming soybeans for over 5000 years, while soybean oil has only recently become a significant element of our diet [4]. Soybeans are the most important legume and the fourth most important crop in terms of worldwide crop production, after rice, wheat, and maize. Soybeans biosynthesize a range of metabolites that are fundamental in crop yield and abiotic and biotic stress tolerance, disease resistance, seed composition, and flavor enrichment. Owing to the rising demand for soybeans, more output is currently required. Although in 2016, around 340 million metric tons of soybeans were grown globally, the production is influenced by a number of factors, including the availability of macro- and micronutrients as well as temperature of the soil [5]. However, it may become more difficult to obtain sufficient crop yields as the climate continues to change dramatically and soil environments are becoming increasingly more stressful to soybeans. To increase soybean harvest volumes mounting factors, ongoing concerns of environmental pressures such as extreme temperatures, salinity, flooding/drought stress, herbicide induction and the devastating effects of several pathogens, such as bacteria, mold fungi, nematodes, and insects on critical yield loss must be addressed [1,2].

Omics-based interdisciplinary approaches facilitate trait modification/optimization, resulting in optimal and precise design breeding [6]. Here we review a brief overview of the application of metabolomics technologies in crop improvement through genetic modification, their potential for future development, and the consequent assessment of food safety. Razzaq and colleagues (2022) describe the present utilization of advanced metabolomics methods coupled with other OMICS approaches that may be used to: examine the complexities of plant genotype-metabolite-phenotype interactions, facilitating metabolomics-assisted plant breeding for exploring the stress-responsive metabolic markers, uncover the hidden metabolic networks associated with abiotic/biotic stress resistance, and facilitate screening and selection of climate-smart crops at the metabolite level [7]. The fundamental idea underlying metabolic editing is to initially identify the precise genes responsible for key metabolic pathways, then to alter one or more genes associated to those networks.

Food safety is one of the main objections to genetically modified (GM) crops, however these objections should be dispelled by employing the present set of metabolomic technologies as part of a food safety evaluation approach and by using reasonable comparators [8]. Clarke and colleagues (2013) highlight the significance of metabolomics in the safety evaluation of GM crops. One of the world’s most extensive GM crops is a glyphosate-tolerant GM soybean type [9]. Garca-Villalba et al. [9] conducted the initial study on the considerable equivalency of GM soybean using a metabolomic method. Glyphosate binds to and inhibits the activity of EPSPS, an enzyme of the aromatic amino acid biosynthesis pathway (shikimate pathway). The inhibition of EPSPS by glyphosate prevents the plant from synthesizing the aromatic amino acids (phenylalanine, tyrosine, tryptophan) required for protein synthesis. However, certain microorganisms possess a glyphosate-resistant form of 5-enolpyruvoylshikimate-3-phosphate synthetase. The variant utilized in genetically modified crops is often obtained from glyphosate-resistant *Agrobacterium tumefaciens* strain CP4 (CP4 EPSPS). The ability to spray glyphosate on fields without affecting the crop significantly increased the ability to manage weeds in the field and ultimately increase the soybean yields [10] A separate study by Alberto and colleagues (2012) discovered that amino acid profiles could be used to examine how glyphosate affected both susceptible and resistant soybean lines. HPLC profiles for ten amino acids (Asp, Asn, Gln, Glu, Gly, His, Leu Ser, Thr, Tyr,) were compared in two near isogenic pairs in four varieties of soybean roots. Multivariate analysis utilizing principal component analysis (2D PCA and 3D PCA) enabled various groups to be identified and differentiated based on the genetic origin of the soybean, indicating the amino acid responses on susceptible and resistant types [11]. The resultant GMO soybean variety A3244, is renowned for its exceptional agronomic traits, including several biotic an abiotic-resistance and high yielding property [12]. This classic example highlights the potential of metabolomics-guided breeding in soybean improvement.

### Relevance as a Multifunctional Crop

Soybean is the most important worldwide legume crop species worldwide owing to its agro-economic and nutritional value, serving as an essential source of protein and oils (40% and 21% content, respectively) for human consumption, livestock feed, industrial biofuel production, and functional foods [2,13,14]. Animal feed accounts for over 85 percent of global soybean protein meal production whereas the soybean crop is mostly farmed for oil production, with only a small percentage of soybeans consumed directly by humans. Owing to its high oil and protein content, soybean is among the world’s most essential crops as it contributes to 56% of all oilseed production worldwide. Soybeans are also high in vitamins, minerals, phospholipids, saponins, isoflavones, flavonoids, oligosaccharides, edible fiber, free sugars, pterocarpans, phytic acids, peptides, and antioxidant compounds [2,15,16,17]. Although phytochemicals in soybeans are present in small amounts and are not required for normal body function, they confer health benefits and aid in the treatment of a variety of diseases, including cancer, arteriosclerosis, osteoporosis, and metabolic syndrome [2,18,19].

Soybean oil is being developed and marketed as a future fuel source, with attempts being undertaken to enhance soybean-derived biodiesel output. In addition, there is ongoing research where soybean protein-based biodegradable materials are being explored to determine the potential as an alternative for plastic synthesis [13]. In addition, owing to the presence of these phytochemicals such as polyphenols and essential oils, soybean leaves have recently been employed in the cosmetics industry and food products [20]. As a result of soybean–*Bradyrhizobium* symbiosis, soybean can meet 50–60% of its nitrogen demand, therefore significantly contributing to soil fertility improvement through biological nitrogen fixation [17,21]. Soymeal, the residue remaining after oil extraction, is a key metabolizable energy source and the world’s number one protein source for animal feed. Soybeans are also utilized for the production of adhesives, inks, building materials, and lubricants [22].

Soybean is therefore a valuable crop for agriculture, industry, and food and thus becoming a more common crop species attributing to its diverse uses, and high demand. By 2050, the world’s population will have doubled, necessitating double the current food output; whereas worldwide soybean production is much below what is required [5]. As a result, the agricultural biotechnology community is placing emphasis on the modification of seed-specific output traits of soybeans [23]. The study of functional genomics has had a significant impact in this regard, providing large-scale biological data that can be used to determine how specific processes in an organism are regulated and controlled, a branch of molecular biology (i.e., metabolomics) that utilizes the huge amount of genomic data available to determine gene functions and interactions [22].

## 2. Metabolomics at the Forefront of Functional Genomic Approaches

**Figure 1:**
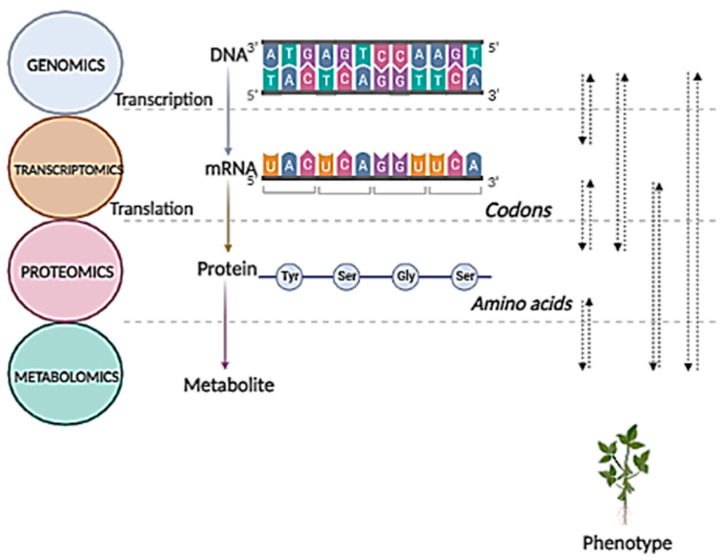
A systems biology perspective on the biological information pipeline. The illustration depicts the integrated flow of biological data via the omics system, from the genome to the metabolome. Metabolomics provides a comprehensive overview of an organism’s biochemical and physiological status, and changed metabolomes reflect changes in the genome, transcriptome, and proteome. As a result, the metabolome is regarded as the underlying biochemical layer that reflects all information expressed and regulated across all the omics layers, providing the most direct relationship to the phenotype. Figure created using BioRender (https://biorender.com/).

Metabolomics refers to a comprehensive modern “omic” approach for analyzing metabolites in a biological system under a specific physiological condition [13,24,25,26,27]. According to the central dogma model, biological information is sequentially transmitted respectively from the genome, transcriptome, proteome, and metabolome (Figure 1). The biochemical phenotype of an organism is represented by the metabolome detail and, thus, a metabolomic investigation unravels the links from the genotype to the phenotype [28,29,30].

**Figure 2:**
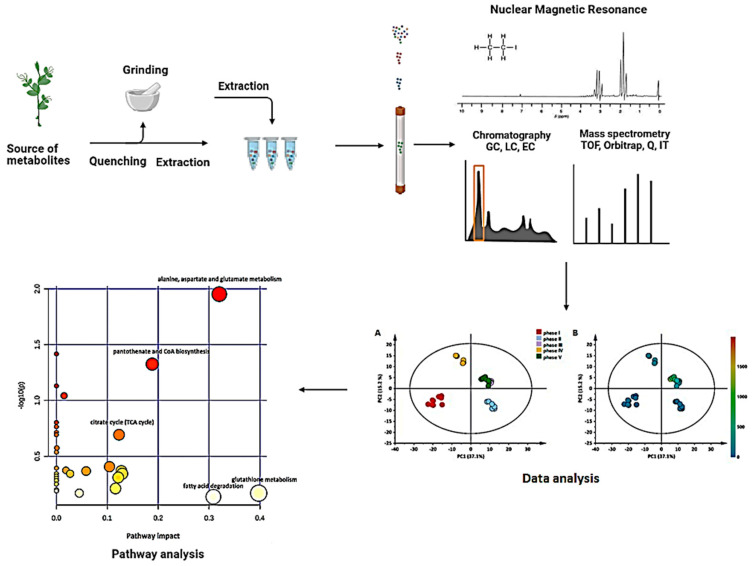
Workflow of a metabolomics approach. Figure created using BioRender (https://biorender.com/).

Metabolomics is a multidisciplinary field that includes biology, analytical chemistry, and multivariate statistics. Three main steps are involved in a metabolomics study: sample preparation, data acquisition, and analysis [31] as illustrated in Figure 2.

### 2.1. Sample Preparation

Sample preparation is the most basic and crucial stage for all plant molecular biological studies since sample integrity dictates the entire outcome of the experiment, i.e., the acquired data and the subsequent biological interpretation. It is critical to keep experimental and biological variance to a minimum to ensure the metabolomic analyses are consistent, robust, and valid [32,33,34]. However, a metabolomic approach generally requires minimal sample preparation relative to the other genomic approaches such as genomics, transcriptomics, proteomics, which are labor intensive [35,36]. However, a diverse set of metabolites with varying physio-chemical complexity and relative abundance poses numerous challenges in plant metabolism [37]. The most common extraction method includes liquid–liquid extraction (LLE), solid-phase extraction (SPE), supercritical fluid extraction (SFE), and microwave aided extraction (MAE) [38,39,40,41,42,43,44,45].

Factors to consider when choosing an extraction technique include selectivity to the widest range of metabolites possible and reproducibility. Regardless of their development, none of these methods can extract the whole metabolome from a biological sample. Each method comes with a built-in bias in favor of a particular class of chemical [37].

### 2.2. Data Acquisition

There has been significant progress in the development of new technologies for metabolomic platforms, which has resulted in the creation of additional data [46]. Several effective analytical platforms are constantly developed and modified in an attempt to comprehensively include as many secondary metabolites as possible. Thus, in plant metabolomics, chromatography and mass spectrometry are the most common techniques used. The invention of ultra-high performance liquid chromatography (UHPLC) heralded the beginning of innovation in LC-based metabolomic platforms by addressing the problem of poor resolution of data gathered using high-performance liquid chromatography (HPLC). However, substantial advancements have been witnessed in the application of Fourier-transform infrared spectroscopy (FTIR), capillary electrophoresis (CE), and nuclear magnetic resonance (NMR) each with its own set of advantages and disadvantages [2,47,48].

The hyphenation of chromatography (liquid and gas-based) and mass spectrometry has proven to be an indisputably efficient platform utilized in metabolomic studies, particularly plant metabolic fingerprinting investigations because a single run can gather data from two functions [49]. The advantages of mass spectrometry in metabolomic analysis are high sensitivity, repeatability, and adaptability. This platform generates an ion by removing or adding a charge from a neutral species, then measures the *m/z* (mass–to–charge ratio) of the ions to provide structural information based on the fragmentation pattern obtained. Prior to entering the mass spectrometer, chromatographic separation of unprocessed biological materials further assists metabolite annotation by adding the retention time (Rt) identifier, increases sensitivity, and decreases signal suppression [50,51].

NMR is a spectroscopic technique that makes use of an atom’s spin characteristics to identify and quantify elements. The method is robust and highly selective, albeit limited by low sensitivity. On the other hand, NMR is unrivaled in the annotation of metabolites due to its capacity to provide the structural intricate details about a molecule. An added advantage of NMR is the capacity to provide semi-quantitative information, as the intensity of the acquired signal is directly proportional to the number of nuclear spins [52,53]. To cover a greater spectrum of metabolites, a full global investigation of an organism’s metabolome frequently necessitates the use of parallel analytical platforms [54].

### 2.3. Data Analysis

Although great strides have been made in the optimization of analytical platforms for data acquisition in metabolomic applications, each platform still has limits. As a result, various steps are conducted post- raw data collection to facilitate metabolite annotation. The initial step is visual data examination followed by data processing and metabolite annotation and, ultimately, biological interpretation [31].

#### 2.3.1. Data Visualization (Pre-Processing and Pre-Treatment)

The visual inspection of graphical information is a vital stage in determining the quality of the raw data and selecting the most appropriate parameters for the successive steps of data processing workflow. However, high-throughput methods generate a large volume of raw data that is not feasible to analyze without automated information technology. As a result, managing these data sets holistically by hand is unfeasible. As a result, a variety of comprehensive software tools and mathematical algorithms for automatic raw data processing have been developed and are all capable of carrying out automated peak picking and other processing functions efficiently [9,55,56,57,58,59]. In this step, raw data are presented in the form of chromatograms and/or spectra as per the various data acquisition mode (Section 2.2).

In the literature, the terms pre-treatment and/or pre-processing are used interchangeably. Essentially, the ultimate purpose of these statistical steps is to eliminate all unrelated factors (experimental and/or analytical) while retaining useful biological information [55,60]. The most common and preferred pre-treatment and pre-processing methods are transformation [9,37], filtration and filling [37,61,62], spectral deconvolution [9,62,63], normalization [24,61], and peak alignment [9,37,61].

#### 2.3.2. Statistical Modelling

The resulting high-throughput raw data matrix is exported to multiple software packages for robust statistical modeling utilizing univariate statistics and multivariate data analysis [9,55]. The most prominent are principal component analysis (PCA), hierarchical cluster analysis (HCA), and partial least squares regression (PLS). PCA is generally the basis for data analysis, wherein pattern recognition model enables the quick display of similarities and differences between sample groups by compressing the multidimensionality of data into a reduced number of variables known as principal components. PCA modeling is thus an unsupervised technique (i.e., without a priori class information) for investigating untargeted metabolic data because it accounts for the overall variance of the dataset provided without the requirement for a priori sample class information [9,64,65,66]. PLS, as a supervised classification model (i.e., with a priori class information), is beneficial when the unsupervised model does not capture the characteristic biomarkers that distinguish between different sample groups. The extension, orthogonal partial least squares/orthogonal projection to latent structures-discriminating analysis (OPLS-DA), explains just the reaction to biological variation, i.e., unique metabolite profiles significantly correlated to the specified response structure [55,66].

### 2.4. Metabolite Annotation, Pathway Mapping, Network Correlation and Biological Interpretation

In metabolomics, the assignment of appropriate metabolite annotation chemical formulas as well as metabolite annotations is a computationally and analytically challenging task. The lack of standardized experimental settings, as well as the biochemical diversity of metabolites, significantly add to the task’s complexity [67]. Significant progress has been made in the advancements of metabolite annotation databases and user-friendly software resources to overcome these constraints. In this regard, there are various free and well-developed software databases available that provide searches based on precise mass and chemical formulas [67,68,69,70,71,72]. However, in some circumstances, annotation of metabolites based only on precise mass and chemical formula may be insufficient. As a result, it is a critical step to back up computationally generated data with experimental evidence. The list of the tentatively identified metabolites are traditionally presented in the form of a table and/or chemical structures.

The biological interpretation of the overall findings is dependent on the correct assignment of the annotated metabolites’ biological roles. Network modeling and pathway mapping tools enable the comprehension of the biological interactions between metabolites. Accordingly, metabolite profiling enables the interpretation of interconnections that arise primarily through metabolic regulation [13].

## 3. Application of Metabolomics as a Prospective Tool to Improve Soybean

Metabolomics advancements enable scientists to rapidly map individual metabolites to the genes that encode their metabolic pathways, providing plant scientists with an exceptional chance to thoroughly study and rationally utilize the plethora of metabolites that plants biosynthesize.

Metabolomics is an effective method for measuring biological or physiological reactions to environmental changes, particularly when combined with other profiling technologies such as transcriptomics and proteomics [21]. Although more robust when combined with other “omics” approaches, the knowledge obtained from metabolomics can contribute to the holistic biological profiling of an organism [13].

Metabolomics has a broad range of applications including the annotation of specific genes [29,65], unravelling metabolic pathways [73], evaluation of biomarker products resulting from transgene expression [65] and environmental perturbation in plants [29], clinical diagnostics of diseases, evaluation of environmental research, drug action research [37], plant taxonomic evaluation [29], biotechnological engineering, food nutritional science [37,73]. In the context of this review, metabolomics advancements enable scientists to rapidly map individual metabolites to the genes that encode their metabolic pathways, providing plant scientists with an exceptional chance to thoroughly study and rationally utilize the plethora of metabolites that plants biosynthesize [7].

As an example of the application of metabolomics, previous studies have reported the adaptive responses of soybean to biotic and abiotic stressors, as well as the major primary and secondary metabolites involved in the adaptation and sensing mechanisms as shown in Table 1.

Although significant literature on critical information about the specific metabolite alternations that occur in response to diverse stress circumstances has been documented (Table 1), plant response to biotic and abiotic stress is a complicated and dynamic process. Most of the current research focuses on a single abiotic stress, although in practice, multiple stresses are commonly present. The interaction of these pressures will influence the physiological response of plants. Furthermore, different organs or cultivars of soybean plants respond differently to the same stress, and the plant’s metabolic system is a constantly changing network of interconnections [2,96,97]. Nevertheless, the study of the soybean metabolome paves the way to a better understanding of complex metabolic pathways and stress-associated metabolites. Metabolomics research can, therefore, pave way for the identification of metabolites as biomarkers of various environmental stressors.

Metabolomics-guided plant breeding programs such as mGWAS (Metabolite Genome-Wide Association Study) and mQTL (methylation quantitative trait locus) mGWAS analysis has proven to be critical for dissecting the genetic and metabolic architecture of rice by finding the genes related with natural variation in rice metabolism [7,90,98,99]. Chen and colleagues (2014) used GWAS to detect 6.4 million SNPs from 529 distinct rice strains, and 36 potential genes that regulate the levels of at least 34 recognized primary and secondary metabolites were identified. Here, this technique is essential for performing molecular phenotypic trait mapping for the purpose of rice improvement. Using flow infusion high-resolution mass spectrometry (FIE-HRMS) [98], Yadav et al. (2021) investigated metabolomic fingerprinting of 197 pearl millet inbred lines and identified numerous metabolite characteristics linked to nutritional benefits such lipid metabolism, vitamins, antioxidants, and dietary starch [99]. The wealth of metabolomic -related research on soybean (Table 1) highlights the potential of such metabolomics-guided plant breeding programs to be utilized toward soybean crop improvement.

## 4. Concluding Remarks and Future Perspectives

Metabolomics, a new and developing field that can predict several biomarkers and characterize the molecular traits involved in physiological processes, is at the forefront of making significant advances in soybean functional genomics research-based methodologies. Thus, the present review provides information on the metabolomics workflow and highlights the prospects of metabolomics in determining key biomarkers associated with mitigating biotic and abiotic stresses to provide valuable information that will guide the soybean breeding programs to produce improved cultivars with value-added features. The principal idea of metabolic editing is to first discover the precise genes responsible for the important metabolic pathways, then change one or more genes connected with those networks. Here, the role of mGWAS in the contribution to the success of genetic modifications and analyses of biomarkers that result in improved soybean yield and stress tolerance has been discussed.

Generating metabolite databases for important crop species under environmental stresses is a time-consuming task. To address these drawbacks, enhancing the resolution and coverage of the metabolome can help to gain a comprehensive understanding of how soybean adapts to biotic and abiotic stress, opening new options for increasing crop yields. Furthermore, although the biochemical and molecular specifics of these pathways are still being worked out, in-depth insights are progressively being achieved through the advancement and development of systems biology strategies. This work provides useful information that may be used in potential metabolic engineering and molecular breeding efforts to improve soybean seed quality and yield in the future. Future investigations may focus on dissecting the metabolome of soybean seeds at different physiological stages, as well as linking the metabolic variations to genomic changes.

## Figures and Tables

**Table 1:** Recent progress in soybean metabolomics studies for identification of key biomarkers to mitigate biotic and abiotic stress tolerance and other growth conditions.

Objective of the Study	Analytical Platform	Tissue	Other Omics	Main Finding	References
**Analyzed the organ specificity of metabolites and identification of the features of their regulatory networks in dehydrated soybeans**	GC-TOF-MS LC-MS	Leaves Stems Roots	Transcriptomics	ABA is the most highly dehydration-inducible phytohormone in plant aerial parts.	[74]
**Investigated metabolite changes in relation to physiological responses two soybean genotypes with varying drought tolerance**	^1^H NMR	Leaves Nodule		Markers important for determining water stress response were identified.	[75]
**Elucidated the mechanism behind drought tolerance in drought-tolerant wild soybean**	GC-MS	Leaves		Drought-stress mechanisms include the accumulation of osmotic chemicals, as well as an increase in energy and secondary antioxidant metabolism. Drought resistance in wild soybeans.	[76]
**Described the metabolic changes in soybean leaves ten days after Soybean mosaic virus infection (SMV)**	LC-MS/MS	Leaves	Transcriptomics	There were significant changes in amino acid concentrations in connection to viral infection at the metabolomic level.	[77]
**Investigated the potential organ-specific resistance mechanism of soybean to *F. Moniliforme***	GC–MS	Seeds Pods		*F. Moniliforme* disrupted amino acid metabolism in soybean seeds, and metabolic pathways involved to energy conversion in soybean pods responded substantially to fungal infection.	[78]
**Examining the responses to flooding stress in roots and leaves of two soybean cultivars (BR4 and Embrapa 45, sensitive and moderately tolerant to flooding stress, respectively).**	^1^H NMR	Roots Leaves		Different reactions were observed in the roots and leaves, as well as in flood-tolerant and flood-sensitive cultivars. The majority of the molecules that have transformed are associated to carbon and nitrogen metabolism, as well as the phenylpropanoid pathway.	[79]
**Two wild soybean types with varying salt tolerance were chosen, and metabolic alterations in response to neutral-salt stress and alkali-salt stress were studied.**	GC–MS	Leaf		The salt-tolerant wild soybean modifies amino acid and organic acid metabolism to generate more suitable solutes and promote the TCA cycle to produce more ATP.	[3]
**Investigated the metabolic changes in soybean cyst nematodes after treatment with Sneb545Bacillus simplex. Roots of SCN-infected soybeans**	GC-MS	Root		Soybeans treated with Sneb545 have certain characteristics of SCN disease-resistant soybeans.	[80]
**Investigated Cd absorption and translocation in two different Cd-accumulating soybean cultivars**	CE-MS	Roots	Proteomics	In the Enrei cultivar under Cd stress, amino acids linked to Cd-chelating pathways are quite active.	[81]
**Investigated drought tolerance in tobacco and soybean plants to unravel metabolic pathways affected by increasing dehydration**	LC-MS LC-MS/MS GC-MS	Root Leaf		In both species, the accumulation of metabolites is strongly linked to the degree of dehydration.	[82]
**Profiled leaf metabolites under control conditions, drought, and heat stress in a controlled setting.**	LC-MS GC-MS	Leaves		Drought and heat stress were found to affect metabolites for various cellular processes which regulate carbohydrate metabolism, amino acid metabolism, peptide metabolism, and purine and pyrimidine biosynthesis.	[83]
**Investigated changes in the metabolic profiles of leaves and roots of soybean (*Glycine max* L.) Seedlings cultivated under normal and excess Mo conditions.**	LC-MS/MS	Roots Leaves		Mo stress induced only lipid metabolism and salicylic acid buildup in leaves, whilst in roots the ascorbate–glutathione metabolism and flavonoid/isoflavone biosynthesis significantly increased.	[84]
**Analyzed of two soybean genotypes at the metabolomic level revealed the mechanism of low-nitrogen tolerance.**	GC–MS	Leaves Roots		In order to tolerate low nitrogen, wild soybean synthesizes favorable secondary metabolites under low-nitrogen stress.	[85]
**Examined metabolomics features of wild soybean under several forms of salt stress to determine salt-tolerant processes in wild soybean in the field**	GC–MS	Roots		Under neutral-salt stress, the salt-tolerant wild soybean showed enhanced amino acid, carbohydrate, and polyol metabolisms, whereas under alkali-salt stress, it showed improved organic acid, amino acid, and tricarboxylic acid metabolisms.	[86]
**Explored the salt tolerance-related mechanisms among Soja, wild soybean, semi-wild soybean, and cultivated soybean under two types of salt stress**	GC–MS	Roots		Carbon and nitrogen metabolism, as well as the tricarboxylic acid (TCA) cycle and receiver operating properties (particularly phenolic substance metabolism) of seedling roots, were critical for salt stress resistance and demonstrated a steady decreasing trend from wild soybean to cultivated soybean.	[87]
**Determined the effects of growth temperature and carbon dioxide enrichment on soybean seed components at different stages of development**	GC–MS	Seeds		CO_2_ (enrichment) treatments significantly changed the composition of early seeds but had little effect on mature seeds. Treatment effects on seed constituents were ranked as follows: Age > Temperature > CO_2_.	[88]
**Characterized the resistance of soybeans to foxglove aphid, *Aulacorthum solani* Kaltenbach, at the metabolite level.**	CE–TOF–MS	Leaves		Differences in the amino acids in the soybean leaves influenced the free amino acids found in the aphids, which might be implicated in aphid resistance.	[1]
**Investigated variations in soybean metabolism in response to *R. solani* infection during early and late disease phases, focusing on the regulation of soybean primary metabolism and oxidative stress tolerance**	^1^H NMR	Leaves	Transcriptomics	In response to *R. solani* infection, significant changes in soybean primary metabolism occurred and metabolite levels involved in redox reactions and ROS signaling were also recorded.	[89]
**Distinguished between genetically modified organisms (Monsanto 89,788 variety) and organic soybeans**	DART-HRMS HPLC-HMRS	Seeds		The most important markers were found to be phosphatidylcholines and sugars.	[90]
**Compared the response mechanisms of wild and cultivated soybean to water stress**	GC–MS	Leaves		Drought tolerance mechanisms included increasing primary metabolism to control osmotic potential, synthesizing desirable secondary metabolites and fatty acids, and maintaining a symbiotic relationship.	[91]
**Explored global metabolomic modifications in low-P-tolerant (Liaodou, L13) and low-P-sensitive (Tiefeng 3, T3) soybean genotypes**	LC-MS	Root		Metabolite profiles of both genotypes differed in their responses as numbers of metabolites were exclusively and differentially regulated within each genotype.	[92]
**Examined the impact of overexpressing OASS on soybean nodulation and nodule metabolome**	LC-MS GC-MS	Nodules		There is a slight decrease in the availability of energy metabolites to OASS overexpressing soybean nodules, which is then offset by the breakdown of cellular components to meet the nodule energy metabolism needs.	[14]
**Evaluated root exudates of two soybean cultivars grown under low-, normal-, and high-K+ conditions**	CE–TOF–MS	Root		Soybean cultivars differ in their capacity to release root metabolites by altering the exudation of certain metabolites for improved adaptability to high- and low-K conditions.	[5]
**Investigated the cellular metabolism-related differences among salt-tolerant wild soybean (W2), salt- sensitive wild soybean (W1) and cultivated soybean (C) in the early flowering stage to reveal the adaptive mechanisms.**	GC–TOF–MS	Leaf		Carbohydrate and organic acid metabolism were relatively greater, while the amino acid content and secondary metabolism level were lower in C than W1	[93]
**Evaluated the metabolic responses of improved (I-1) and unimproved (UI-4) soybean genotypes after AM root colonization**	GC-MS	Roots		The I-1 genotype has lower quantities of isoflavonoids and alpha-tocopherol and greater levels of malondialdehyde, that can affect the soybean-AM symbiosis.	[94]
**Investigated secondary metabolites produced when soybean plants were infected by *A. Besseyi*.**	LC–ESI–MS–MS	Root		There were metabolome variations in root defensive chemicals in response to *A. Besseyi* attack, as indicated by an increase in the level of flavonoids.	[68]
**Identify metabolic changes in soybean roots treated with rhizobia inoculation and salt**	LC–TOFMS	Root	Phosphoproteomics	Rhizobia symbiosis enables the soybean plant to adapt with the negative consequences of high soil salt, mostly by increasing ROS scavenging activities.	[95]

## Data Availability

Not applicable.
